# OSA in patients with head and neck cancer is associated with cancer size and oncologic outcome

**DOI:** 10.1007/s00405-020-06355-3

**Published:** 2020-09-29

**Authors:** Tilman Huppertz, Vera Horstmann, Charlotte Scharnow, Christian Ruckes, Katharina Bahr, Christoph Matthias, Haralampos Gouveris

**Affiliations:** 1 Sleep Center of the Department of Otorhinolaryngology- Head and Neck Surgery, University Medical Center of the Johannes Gutenberg University Mainz, Langenbeckstr. 1, 55131 Mainz, Germany; 2 Interdisciplinary Center for Clinical Trials (IZKS), University Medical Center of the Johannes Gutenberg University, Mainz, Germany

**Keywords:** Obstructive sleep apnea, Head and neck cancer, Quality of life, Cardiorespiratory home sleep apnea testing

## Abstract

**Purpose:**

Obstructive sleep apnea (OSA) is associated with severe daytime sleepiness and reduced quality of life. These symptoms are also present in patients with squamous cell carcinoma of the head and neck (SCCHN) before, during and after treatment, so that comorbidity cannot be excluded. The aim was to evaluate the prevalence of OSA and its impact on the quality of life in patients with oropharyngeal, hypopharyngeal and lateral tongue SCCHN in a prospective study.

**Methods:**

We performed cardiorespiratory home sleep apnea testing and recorded sleep-related patient-reported outcomes in 33 patients with confirmed oropharyngeal, hypopharyngeal and lateral tongue SCCHN. We correlated the sleep-related variables to oncologic variables and endpoints.

**Results:**

Five female and 28 male patients with SCCHN (aged 46–77 years) were recruited. Thirty patients (90%) had OSA as defined by an Apnea/Hypopnea Index (AHI) > 5 /h before treatment. Evaluation after treatment, which was possible in 17 patients, showed OSA in 16 patients (94%). Radiologic primary tumor size showed significant positive correlation with AHI and apnea-index. Tumor recurrence and tumor-related mortality showed significant positive association with AHI. PSQI of these patients showed at least a moderate sleep disturbance. EORTC QLQ c30 questionnaire showed reduced values for all tested qualities, in particular for fatigue, insomnia, pain and financial distress.

**Conclusion:**

Obstructive sleep apnea is a significant comorbidity in patients with SCCHN. Pre-interventional AHI may be correlated with the oncologic outcome. Further research is needed to further describe the course of OSA and its treatment before, during and after therapy.

## Introduction

Obstructive sleep apnea (OSA) affects almost 50% of the male and almost 25% of the female adult population in Western Europe [1], with similar figures worldwide [2]. It may be associated with daytime sleepiness, missing sense of recovery after sleep and fatigue. OSA is independently associated with an increased risk of developing metabolic syndrome, hypertension, incident coronary heart disease or heart failure, diabetes, stroke and depression [1, 3–8]. Sleep-disordered breathing is associated with increased cancer mortality in a community-based sample and a higher incidence of breast cancer [9, 10].

Squamous cell carcinoma of the head and neck (SCCHN) is the seventh most common cancer worldwide and the third most common cause of cancer-related death in the world with over 500,000 new cases reported annually [11–15]. Smoking, alcohol use, smokeless tobacco use, and HPV infection are the major risk factors for oral cavity cancer, with smoking and alcohol having synergistic effects [14].

Head and neck cancer and its treatment lead to a wide variety of symptoms that decrease the quality of life of these patients. Among these are also symptoms that could in part be explained by concomitant OSA [16–18].

Some studies have reported a coexistence of head and neck cancer and OSA. Friedman et al. [19] reported an OSA-incidence of 91.7%, however the measurement was performed after the successful treatment of a mixed group of 24 patients with cancer of base of tongue, pharynx-und larynx. Faiz et al. [16] reported an OSA-prevalence of 88% in head and neck cancer patients also after radiochemotherapy. On the other hand, Nesse et al. [20] reported a post-interventional OSA-prevalence of only 12% in a population of SCCHN patients with primary malignancies localized in the oral cavity and oropharynx. Steffen et al. [21] similarly could not find any significant correlation between radiotherapy and prevalence of OSA in SSCHN patients.

In one prospective cohort study performed after cancer treatment in patients with advanced oropharyngeal cancer (AJCC Stage III or IV) and treated with radiotherapy or surgery, a concomitant OSA was associated with a significant decline in Global Health Status Scale as well as a significant increase of the fatigue item of the EORTC QLQ C-30-questionaire [17]. In another prospective study in 17 patients by Payne et al. (2005, [22]) 76% had an AHI greater than 20 /h with a mean AHI of 44.7 /h before therapy.

Therefore, there is evidence of existence of comorbid SCCHN and OSA. The aim of this study was to generate prospective data on the concurrence of head and neck cancer with OSA before initiation of oncologic treatment as well as prospective data on sleep-related quality of life in these patients.

## Materials and methods

The study was approved by the local ethics committee (Nr. 20114566 of local ethics committee approval). All patients gave signed informed consent for their participation in the study. 110 consecutive patients with confirmed head and neck cancer were screened prior to the treatment from January 2018 to June 2019. 33 of these patients gave informed consent and were included in the study and 17 of them consented to a second screening after treatment. Cancer localization included tongue, oropharynx and hypopharynx. Primary cancer T-stage included cT2–cT4 tumors based on clinical and radiologic criteria. Cardiorespiratory home sleep apnea testing (HSAT) was performed using the MiniScreenPlus^®^ device (Loewenstein Medical, Bad Ems, Germany). HSAT—signals included respiratory flow via a nasal cannula pressure transducer, thoracic and abdominal respiratory effort via inductance belts, SpO2 and heart rate via pulse oximetry and body position recording. As apnea was defined any amplitude reduction of the nasal airflow signal greater than 90%, which lasted for at least 10 s. As hypopnea was defined any reduction of the airflow signal on the nasal flow sensor between 30 and 90% of pre-event baseline for ≥ 10 s with an associated ≥ 3% reduction of the peripheral arterial blood oxygen saturation (SpO2). Patients were also asked to fill out the Epworth sleepiness scale (ESS), the Pittsburgh Sleep Quality Index (PSQI), the Insomnia Severity Index (ISI), the EORTC QLQ-C30 version 3.0 and the SF 36 questionnaires. In PSQI each one of seven components may be assigned a value of 0–3 and an increasing total score (range 0–21 points) is evidence of decreasing overall sleep quality. In ISI the score may range for each one of the seven components from 0 to 4 and an increasing total score suggests a more severe insomnia; values higher than 15 correspond to clinical insomnia.

EORTC includes five functional scales, three symptom scales, a global health status/QoL scale and six single items. Scales and single-item measures range in score from 0 to 100. A high scale score in the functional scales represents a better level of function whereas a higher score in the symptom scales and single items represents stronger symptoms.

In SF36 there are eight scales (Physical functioning, Role limitations due to physical health, Role limitations due to emotional problems, Energy/fatigue, among others) and 36 question items. The higher the total scores, the better the general quality of life of the responder.

### Statistical analysis

For statistical purposes analysis of covariance models with tumor localization and Union Internationale Contre le Cancer (UICC)-staging as fixed factors and age, BMI (body mass index), neck circumference, tumor size and baseline AHI as random effects were fitted to investigate the influence on sleep-related parameters. Pre-/post- treatment values were compared by a one-sample *t*-test of the change difference. To quantify potential effects, correlation analyses and descriptive statistics were conducted. Although all analyses were done purely exploratory, a *p* < 0.05 was considered as significant. The analyses were performed on all available data. Missing values were not replaced. All analyses were performed by means of SAS, Version 9.4 (SAS Institute Inc, Cary, NC).

## Results

Of the 33 patients included, six were female (18%), 27 male (82%). They were aged 46–77 years (median 64 years). Body mass index ranged from 15.8 to 31.4 kg/m^2^ with an average of 24.6 kg/m^2^. Cancer stage (UICC) of included patients ranged from I to IV B. Of these 33 patients, 12 received primary radio-chemotherapy (RCT) and 21 received surgery followed by adjuvant radiotherapy (RT) (15 patients) or RCT (six patients). Two patients in the adjuvant therapy group withdrew from therapy after receiving 48 and 18 gy.

Out of the 33 participants, 30 (90%) had obstructive sleep apnea as defined by an Apnea/Hypopnea Index (AHI) > 5 /h. Of these, 18 (54%) had mild (AHI 5–15 /h), 8 (24%) had moderate (AHI 15–30 /h) and 4 (12%) had severe (AHI > 30/h) obstructive sleep apnea. 45.5% had supine position-related sleep apnea, defined by an overall AHI greater than 5 events/h, and a supine AHI greater than double the non-supine AHI [23]. Median oxygen saturation was 92.3% (Table 2). Further demographic data can be seen on Table 1.

**Table 1:** Pre-treatment characteristics of patients included

Gender male	81.8%
Age, years	64.42 (± 7.83)
BMI before treatment, kg/m^2^	24.83 (± 3.63)
BMI after treatment, kg/m^2^	22.82 (± 3.76)
Neck circumference cm	39.38 (± 3.84)
Arterial hypertension	56.3%
Diabetes mellitus	18.2%
History of other previous malignancy	6.1%
Smokers	66.7%
Daily alcohol consumption	39.3%

Presented are the pre-treatment characteristics of the participants. Absolute numerical values correspond to the mean values, with standard deviations within parentheses

Primary tumor size (in cm) on head and neck CT scans showed significant positive correlation with AHI and apnea-index. Clinical outcome (recurrent cancer or tumor-related mortality) was also significantly associated with higher AHI, with a median AHI of 18.82 /h in patients without recurrent disease or tumor-related mortality and a median AHI of 22.10 /h in patients with recurrent disease or tumor-related mortality.

We included 17 patients for a second round of cardiorespiratory HSAT after completion of oncologic treatment. In this group of participants we found a median AHI of 20.4 /h with a range from 4 to 36.1 /h. Furthermore, 16 of the 17 participants (94%) had an AHI > 5 /h. Median desaturation index was 17.8 /h and total sleep time (TST) spent with arterial oxygen saturation (SaO2) < 90% (T90) increased before treatment and returned to normal values after treatment (Table 2).

**Table 2:** Sleep parameters and sleep-related questionnaires

	Before treatment	After treatment	*P* (*t* test)
AHI (events /hour)	20.24 (± 15.49)	20.49 (± 11.95)	0.9519
Apnea index (events/hour)	10.97 (± 12.33)	10.34 (± 10.43)	0.9520
Hypopnea index	9.26 (± 6.12)	10.15 (± 6.38)	0.9733
Desaturation index (events/hour)	18.48 (± 12.87)	17.76 (± 10.81)	0.8360
O_2_ saturation, %	92.39 (± 6.51)	94.47 (± 1.59)	0.0069
t 90%	8.48 (± 15.18)	2.31 (± 2.86)	0.0987
Epworth sleepiness scale (ESS)	7.61 (± 4.85)	9.35 (± 4.03)	0.1846
Pittsburgh Sleep Quality Index (PSQI)	9.19 (± 4.57)	10.12 (± 4.44)	0.2168
Insomnia Severity Index (ISI)	12.14 (± 6.84)	12.88 (± 6.18)	0.5626

Depicted are the cardiorespiratory HSAT metrics mean values (± standard deviation) as well as the patient-reported sleep-related outcomes (also mean values ± standard deviations) based on standard questionnaires

Table 3 depicts the mean difference in AHI numerical values before and after treatment (hence negative values meaning AHI increase after treatment) in relation to UICC staging which shows that the higher the UICC-stage, the greater the AHI reduction that was observed after therapy. This is irrespective of mean age, BMI or neck circumference and shows that in patients with small cancers the therapy-/radiation- induced changes tend to lead to development of apneas whereas in large cancers reduction of cancer volume during therapy leads to less apneas.

**Table 3:** Sleep-related, anthropometric and radiomorphologic size data of the study participants based on UICC-stage

UICC Stage	Pre-treatment AHI	Treatment-associated mean difference in AHI	Mean age in years	Mean BMI	Mean neck circumference in cm	Mean cancer size in cm on CT-scan
1 (*N* = 2)	45.35 (± 0.35)	− 16.25 (± 30.62)	63.00 (± 2.83)	26.35 (± 1.06)	42.00 (± 0.00)	3.90 (± 1.56)
2 (*N* = 8)	18.11 (± 20.60)	− 1.46 (± 19.06)	58.38 (± 6.97)	24.50 (± 2.90)	39.88 (± 3.87)	3.38 (± 1.78)
3 (*N* = 3)	23.90 (± 21.42)	0.37 (± 11.57)	67.00 (± 8.19)	26.77 (± 4.86)	41.00 (± 4.58)	3.17 (± 1.06)
4 (*N* = 4)	11.35 (± 9.80)	9.68 (± 7.87)	62.25 (± 3.30)	21.98 (± 4.48)	36.25 (± 3.59)	4.85 (± 2.10)
*p* values	0.2506	0.8284	0.8768	0.6625	0.2874	0.5261

Depicted are the sleep-related, anthropometric and radiomorphologic head and neck computed tomography (CT) size data of the study participants based on UICC-stage. All values represent means with the respective standard deviations within parentheses. Group comparisons were done using the *F* test

In Table 4 the difference in mean AHI, this time depending on localization of primary cancer, is shown. Oropharyngeal cancers do not show much difference in AHI before or after therapy, hypopharyngeal cancers on the other hand present a few apneas less after treatment.

**Table 4:** Sleep-related, anthropometric and radiomorphologic size data of the study participants based on primary tumor localization

Localization of primary cancer	Pre-treatment AHI	Treatment-associated mean Difference in AHI	Mean Age in years	Mean BMI	Mean neck circum-ference in cm	Mean cancer size in cm on CT-scan
Hypopharynx (*N* = 3)	12.33 (± 2.01)	6.77 (± 11.33)	64.67 (± 1.53)	27.0 (± 4.45)	42.67 (± 2.08)	3.13 (± 1.39)
Oropharynx (*N* = 14)	22.55 (± 20.68)	− 1.76 (± 18.43)	60.64 (± 7.04)	23.99 (± 3.43)	38.79 (± 3.95)	3.88 (± 1.80)
*p* values	0.2506	0.5831	0.8768	0.6625	0.2874	0.5261

Depicted are the sleep-related, anthropometric and head and neck computed tomography (CT) size data of the study participants based on primary tumor localization. Although there were initially nine patients with malignancy of the hypopharynx, five with malignancy of the tongue and 19 patients with malignancy of the oropharynx, only 17 patients came to post-interventional follow-up. All values represent means with the respective standard deviations within parentheses. Again, group comparisons were done using the *F* test

The Pittsburgh Sleep Quality Index (PSQI) of these patients showed elevated results, which did not change after therapy. The same was true for Insomnia Severity Index (ISI), which showed subthreshold insomnia that did not change with treatment. Epworth sleepiness scale (ESS) score ranged from 1 to 18 with a median of 7.6 before and a median of 9.3 after treatment (Table 2).

Table 5 depicts mean EORTC values of this cohort in comparison to general population data, showing reduced values in the functioning scale and elevated values in the symptom scale with especially high values for items of fatigue, insomnia and pain [24]. Mean values for insomnia, pain and financial difficulties were even higher after therapy.

**Table 5:** EORTC norm values and cohort values

	EORTC norm values [24]	Before therapy	After therapy	*p* value (*t*-test)
Functioning scale				
Global health status/QoL	75.70	54.60 (± 25.74)	42.36 (± 20.24)	0.1291
Physical functioning	91.00	79.76 (± 19.73)	73.90 (± 23.68)	**0.0261**
Role functioning	88.10	72.87 (± 33.13)	54.17 (± 29.42)	**0.0124**
Emotional functioning	83.20	59.71 (± 26.84)	62.50 (± 23.70)	0.4149
Cognitive functioning	90.50	79.31 (± 21.66)	70.83 (± 17.58)	0.1889
Social functioning	91.50	72.42 (± 24.51)	54.17 (± 35.62)	0.0241
Symptom scale				
Fatigue	19.50	36.31 (± 27.62)	52.78 (± 26.02)	0.0666
Nausea and vomiting	3.10	4.60 (± 18.85)	16.67 (± 33.34)	0.1195
Pain	16.50	44.58 (± 37.89)	36.12 (± 30.03)	0.6838
Dyspnea	11.10	27.59 (± 35.72)	22.23 (± 29.60)	0.7776
Insomnia	15.70	41.38 (± 40.49)	49.99 (± 36.25)	0.6385
Appetite loss	4.80	12.64 (± 24.25)	33.33 (± 40.21)	**0.0339**
Constipation	5.20	14.94 (± 26.10)	16.67 (± 26.60)	1.0000
Diarrhea	4.90	13.79 (± 20.92)	8.33 (± 20.72)	0.0819
Financial difficulties	5.70	20.68 (± 30.10)	66.70 (± 34.84)	0.2693

Presented are the EORTC norm values [24] as well as the cohort values before and after therapy. The numbers represent the mean values (± standard deviation). Pre-/post- treatment comparisons were done using t-test. A *p* value less than 0.05 was statistically significant

The insomnia item of the EORTC QLQ c30 correlated significantly with PSQI (Pearson 0.87, *p* < 0.001), ISI (Pearson 0.62, *p* = 0.011) and desaturation index (Pearson 0.50, *p* = 0.03) before therapy. The fatigue item of the EORTC QLQ c30 on the other hand correlated to radiologic tumor size (Pearson 0.78, *p* = 0.02) but not to the other sleep quality questionnaires. Other correlations of these two items with nightly respiratory parameters such as AHI or cancer parameters were tested but were very weak and did not reach significance.

Table 6 shows results of SF36 questionnaire for the male patients comparing them to norm values of a German standard population [25]. Here as in the EORTC a general reduction in quality of life is documented, which for the fatigue scale is worse after treatment than before. Since standard values are documented only by gender and we only had six female subjects in the study population, we only included males in the evaluation of SF 36.

**Table 6:** SF 36 norm values for males and values in the study cohort

	SF-36 norm values (men) [25]	Before therapy	After therapy	*p* value (*t* test)
Physical functioning	88.18	71.00 (± 28.06)	63.75 (± 25.86)	**0.0385**
Role limitations due to physical health	85.53	42.70 (± 42.66)	64.58 (± 45.80)	0.0915
Role limitations due to emotional problems	91.58	48.47 (± 48.28)	75.00 (± 45.23)	**0.0291**
Energy/fatigue	62.58	54.42 (± 22.35)	40.42 (± 20.17)	**0.0047**
Emotional well-being	75.22	67.52 (± 19.78)	62.33 (± 29.43)	0.4659
Social functioning	88.63	70.90 (± 27.88)	53.13 (± 29.74)	**0.0243**
Pain	71.04	54.74 (± 28.54)	46.25 (± 30.22)	0.5278
General health	66.83	48.96 (± 17.07)	42.92 (± 19.12)	0.5364

Presented are the SF36 norm values for males [25], given that the vast majority of the participants were males, as well as the cohort values before and after therapy. The numbers represent the mean values (± standard deviation). Pre-/post-treatment comparisons were done using *t*-test. A *p*-value less than 0.05 was statistically significant

## Discussion

In this study, we provide for the first time prospectively gathered evidence for existence and associations of comorbid OSA and SCCHN already at the time of first SCCHN diagnosis. The vast majority of patients with SCCHN in this study population have comorbid OSA before as well as directly after treatment. Furthermore, cancer size as measured on CT scan correlated positively with both AHI and apnea index, suggesting that apneas, rather than hypopneas, make up the most relevant part of OSA in this specific patient group. We also found that cancer stages according to UICC are associated with therapy-associated AHI changes; more specifically, patients with small primary cancers tend to develop more apneas and hypopneas during treatment whereas patients with large cancers have less apneas and hypopneas after treatment. This may be due to the fact that the therapy-associated reduction of cancer volume far outperforms the OSA-promoting effect of radiotherapy-induced mucosal edema and the functional/structural compromise of the cranial nerves and musculature. Possibly, quite the opposite may be true in small cancers. Also clinical oncological outcome, as defined by recurrent disease or cancer-related mortality, was significantly associated with AHI. In our study cohort mean BMI was found to be normal, despite a high AHI; this excludes BMI as a confounding factor for the extremely high prevalence of OSA in this patient population and strongly suggests that OSA is the result of the underlying cancer disease or its treatment. T90 returned to normal values after therapy, maybe as a result of oncologic therapy-induced weight loss, as documented by the post-therapeutic BMI reduction in our patient cohort. Sleep-related quality of life was already reduced in this group before any treatment and did not significantly change after therapy, as documented by PSQI values of 10 or ISI score of 12. EORTC QLQ c 30 questionnaire shows reduced quality of life scores for almost all tested properties, in comparison to the general population, with high values for fatigue and insomnia. Mean values were even more elevated for these two items (namely fatigue and insomnia) after therapy. Significant correlations could be established for the insomnia item of the EORTC and PSQI, ISI and desaturation index as well as for the fatigue item and radiologic tumor size. SF-36 data also show reduced quality of life scores.

### Strengths of the study

This prospective study was performed in a cohort of patients with defined tumor location, clinical standard staging and treatment. Home sleep studies were carried out for all patients in the same manner and according to current clinical standard. OSA risk factors (BMI, age) were analyzed in the study population.

### Limitations of the study

This was an exploratory study, hence no formal sample size calculation was performed. Although the number of participants who completed the study was small (17 patients), the study has still a power of 80% to detect pre-/-post differences of an effect size of 0.73. Another limitation include the high number of initial drop-outs not willing to participate in the study which predisposes our study to a selection bias potentially including more patients with sleep problems who were more willing to participate due to their pre-existing sleep complaints. Most of these patients did not give informed consent, expressing concern that they would not be able to sleep as accustomed with the diagnostic device. Also almost half of patients could not be included for a second round of evaluation, limiting the statistical power of the observations after treatment.

### Comparison with the existing literature

The prevalence of OSA found in our study population is in line with some of the previously published results [16, 19, 22] and higher than in other reports [17, 20]. It is also higher than published results in the general population [1]. These previous reports were mostly performed retrospectively and looked at OSA and sleep-related quality of life after treatment of SCCHN had been completed. None of the so far published studies, to our knowledge, looked at OSA prevalence and sleep-related quality of life before treatment and in the acute phase after treatment.

Similar to a previous study [19], mean BMI was normal, indicating that apneas in this population are due to the underlying cancer and its treatment and not related to obesity, as is the case in the general population.

The finding that cancer size and UICC stage are positively associated with AHI may be clinically explained, since greater cancer mass leads to greater obstruction in the pharynx. Surgery and radiation therapy lead to shrinking cancer mass but on the other hand cause tissue fibrosis of the dilator muscles of the pharynx, compromising functionally and structurally the cranial nerves as well as causing pharyngeal mucosal edema that may lead to less airway patency during sleep after treatment. The finding that especially large tumors, as defined by high UICC stage, have a reduction in AHI after treatment also shows that OSA in these patients is cancer-related. On the other hand, the mean AHI increase in patients with small cancers shows that OSA emergence through therapy by the above mentioned mechanisms is a possibility. Supine positional OSA was found in 45.5% of the study population before treatment, which ranges in the upper values of previously reported numbers for non-SCCHN OSA-patients [23]. This can also be explained by the fact that cancer mass in the pharynx and enlarged cervical lymph nodes lead to obstruction and facilitate pharyngeal collapse when lying in supine position.

Interestingly, worse clinical oncological outcome, as defined by recurrent disease or cancer-related mortality, was also significantly associated with higher AHI. Recurrent cancer or cancer-related mortality was seen in five patients. Due to the small sample size, this finding should be interpreted with caution. Tumor hypoxia is a well-known cause of radio-resistance in SCCHN [26]. As a result, tissue hypoxia resulting from OSA in SCCHN patients may cause radio-resistance and therefore worse therapeutic outcome. Hyperbaric oxygen therapy is regarded as an efficient adjuvant therapy in the treatment of SCCHN [26]. Additionally, chronic intermittent hypoxia enhanced proliferative and migratory properties of tumors in mouse models of lung cancer [27, 28]. These findings might, at least in part, explain the deleterious effect of more severe OSA on oncologic outcome in SCCHN-patients.

Quality of life and sleep quality, as measured by a variety of questionnaires (PSQI, ISI, EORTC QLQ c30, SF 36), show a decrease that is exacerbated after therapy for most items measured. This is in line with other studies concerning the EORTC QLQ c30 questionnaire, although the reported absolute numbers are higher in this study [29, 30]. Regarding SF 36 similar results have been reported for a general OSA population [31].

## Conclusion

This study underlines the necessity to address more frequently OSA as a cause of fatigue and reduced quality of life in patients with SCCHN in the future.

Additionally, our findings could open the way for a novel treatment approach according to which patients with SCCHN and comorbid OSA may be treated with positive airway pressure (PAP) therapy or to enhance sleep-related quality of life and possibly even oncologic outcome in this specific patient group. To this end, further studies are necessary. After completion of definitive oncologic therapy, mandibular advancement devices may be used as a long-term therapy of post-interventional OSA, as shown in a rabbit model [32].
